# The impact of fellowship training on pathological outcomes following radical prostatectomy: a population based analysis

**DOI:** 10.1186/1471-2490-14-82

**Published:** 2014-10-23

**Authors:** Jasmir G Nayak, Darrel E Drachenberg, Elke Mau, Derek Suderman, Oliver Bucher, Pascal Lambert, Harvey Quon

**Affiliations:** Section of Urology, Department of Surgery, University of Manitoba, Winnipeg, Manitoba Canada; CancerCare Manitoba, Winnipeg, Manitoba Canada

**Keywords:** Education, Fellowship, Pathology, Prostatectomy, Prostatic neoplasms

## Abstract

**Background:**

Radical prostatectomy (RP) is a common treatment for prostate cancer (PCa). Morbidity, mortality and pathological outcomes may be superior in academic institutions. One explanation may be the involvement of oncology fellowship trained urologists within academic institutions. The literature examining pathological outcomes often lacks individual surgeon data. The objective of this study was to compare pathological outcomes following RP between fellowship trained and non-fellowship trained urologists.

**Methods:**

Population-based, retrospective chart review of men diagnosed with PCa between 2003 and 2008, the majority treated with open approach RP (>99%). Pathological outcomes were compared between oncology fellowship trained academic (FTA), non-fellowship trained academic (NFTA) and non-academic (NA) urologists. Relationships with pathological outcomes were examined utilizing multivariable logistic regression.

**Results:**

83.1% of eligible patients were included in our analysis resulting in 1075 patients. In multivariable analysis, surgeon group was an independent predictor of positive surgical margin (PSM) (p < 0.0001). NFTA and NA urologists were more likely to have PSM compared to FTA urologists (OR 2.50; 95% CI: 1.44 - 4.35 and OR 2.10; 95% CI: 1.53 - 2.88, respectively). However, the proportion of PSM between NFTA and NA urologists was not significant (p = 0.492). In addition, pathological stage (p = 0.0004), Gleason sum (p < 0.0001), and surgeon volume (p = 0.017) were associated with PSM. Limitations include retrospective design and lack of clinical and functional outcomes.

**Conclusions:**

Uro-oncology fellowship trained surgeons had significantly lower rates of PSM than non-fellowship trained surgeons in this population based cohort. This study demonstrates the importance of surgeon-related variables on pathological outcomes and highlights the value of additional urologic oncology fellowship training.

## Background

Prostate adenocarcinoma (PCa) is a prevalent disease with an estimated 256,600 cases expected to be diagnosed in Canada and the US in 2014 alone [1, 2]. A significant proportion of patients diagnosed with PCa will undergo a radical prostatectomy (RP). Although a complex issue, it is generally believed that hospitals and surgeons with increased caseloads have reduced rates of post-operative complications, including lower urinary complications and improved oncological outcomes [3–12]. Furthermore, numerous clinical and pathological factors have been shown to be associated with disease recurrence following RP including clinical stage, biopsy Gleason sum, final Gleason sum, pre-treatment prostate-specific antigen (PSA) and surgical margin status [13, 14]. Positive surgical margins (PSM) are associated with increased risks of biochemical recurrence after RP and currently represent a potentially modifiable variable to improve oncological control [7, 13, 15–18]. Furthermore, PSM is one of the very few quality indicators of surgery. It has previously been shown that substantial variation exists in PSM rates between individual surgeons. Even among experienced surgeons, others have shown that PSM rates range from 10-48% in men with organ confined disease, suggesting that the individual surgeon may be an independent risk factor for PSM [5, 7].

Earlier studies have demonstrated that after adjusting for annual hospital caseload, better outcomes were achieved following RP in academically affiliated institutions [19]. Specifically, RP performed in academic institutions were associated with fewer blood transfusions, fewer post-operative complications and shorter lengths of stay in hospital [20]. The authors postulated that this might be due to increased caseloads, continual peer-review through the decision-making process and/or multi-disciplinary team approaches to patients. Unfortunately, this study was limited by lack of individual surgeon data and information on clinic-pathological outcomes. Another potential reason for superior outcomes may be the involvement of uro-oncology trained clinicians within these institutions. Generally speaking, supporters of sub-specialization claim that fellowship training translates into improved outcomes. Urologic oncology fellowship programs provide intensive training with a concentrated surgical experience focusing on oncological theory and skills. In fact, it has been shown in a small prospective, cohort study, that fellowship training can abbreviate the learning curve associated with RP [21]. However, to the authors’ knowledge there have been no studies investigating the impact of oncology fellowship training on pathological outcomes following RP in a population-based cohort.

It is well documented that PSM have negative prognostic implications including increased rates of biochemical recurrence and disease progression [7, 13, 15–18]. Thus, PSM may be used as a surrogate measure of oncological outcomes. As such, we sought to determine the impact of urological oncology fellowship training on PSM rates following RP.

## Methods

The study was approved by the University of Manitoba Research Ethics Board. As this was a retrospective study, informed consent was not obtained from patients.

### Study population

We performed a retrospective population-based study, utilizing a provincial cancer registry to identify all men who were diagnosed with PCa between 2003 and 2008 in whom the vast majority were treated with open approach RP (>99%). A small number of RP’s were performed laparoscopically (estimated, approximately <10) and were included within this study cohort. Robotic assisted RP were not available at the time of our study. All malignancies are mandatorily reported to the provincial cancer registry. The pathological records of these patients are stored in a central location and were reviewed manually.

### Data collection

For all patients, age, year of diagnosis of prostate cancer, year of surgery, surgeon characteristics, and pathological outcomes following RP were obtained. Surgeons were classified as either: fellowship trained academic (FTA), non-fellowship trained academic (NFTA) or non-academic (NA) urologists based on the highest level of training achieved. For the purpose of our study, fellowship training refers exclusively to surgeons who completed accredited Urologic Oncology programs according to the Society of Urological Oncology. Academic urologists who completed other fellowships (e.g. endourology) were considered as ‘non-fellowship’ for the purpose of this study. A hospital was considered an academic centre if associated with an accredited residency training program. There was no crossover between surgeons and their respective institutions. No non-academic surgeons had accredited oncology fellowship training. Pathological reports were reviewed and the following variables were identified: Gleason sum, margin positivity, and lymph node status. Positive margins were defined as cancer at the inked resection margin.

### Statistical analysis

The primary purpose of our study was to evaluate the relationship between the PSM rates following RP and surgeon training. Surgeons were grouped into three categories as previously described (FTA, NFTA, and NA). Age was analyzed as a continuous variable. Annual volume was not linearly related to the outcome. As such, annual surgeon volume was analyzed as a categorical variable, defined as the average number of cases per year over the study period (low: <10 cases/year, medium: 10–20 cases/year, high: >20 cases/year). As these were patients diagnosed with PCa between 2003 and 2008, the patients may have undergone their treatment in a different year than they were diagnosed. Thus, the annual volume was assessed over 2004–2008 as a representation of the surgeons RP practice. Pathological outcomes that were analyzed as categorical variables included: Gleason sum (≤6, 7 (3 + 4, 4 + 3), ≥8) and stage (pT2, pT3a, pT3b, pT4). Nodal status was categorized as presence or absence of pathologically involved lymph nodes.

Logistic regression was conducted to examine factors related to positive margins following radical prostatectomy. The surgeon group variable (FTA, NFTA, NA), as well as the covariates of surgeon volume, pathological stage, Gleason sum, and node status were examined as potential predictors of PSM. Associations between predictor variables of interest and PSM were initially evaluated with univariable models. Variables were considered significant and eligible for inclusion in a multivariable model if p-values ≤0.2. This p-value was chosen to help prevent the inadvertent exclusion of variables whose effect may be masked by another variable (i.e. the effect of a predictor only becomes apparent after controlling for a confounder). Correlation between variables was assessed by Spearman’s Rank correlation test, variance inflation factor and tolerance statistics. Correlation coefficients ≥0.80, variance inflation factor values ≥10, and/or tolerance values ≤0.2 were considered indicative of multi-colinearity. All significant, non-correlated predictor variables were considered for inclusion in a multivariable model using a manual forward, then backward selection. Variables were considered significant if p-value ≤0.05. All model building analyses were performed with Stata statistical software (version 11.0; Stata Corp, College Station, TX).

## Results

Between 2003 and 2008, 1294 patients were identified as meeting our study criteria. 1080 patients were ultimately deemed eligible and included in descriptive analyses, while incomplete pathological reports led to a small number of additional exclusions. The subsequent multivariable modeling was base on 1075 patients, representing 83.1% of all eligible RP’s. Baseline characteristics of patients undergoing RP are in Table 1. Fifteen surgeons were divided according to their fellowship training and academic affiliation resulting in three groups: FTA (n = 2), NFTA (n = 3) and NA (n = 10). The average number of annual RP’s per group was: FTA 20.5 (range: 13.4-27.6), NFTA 4.3 (range: 0.2-11.4) and NA 12.1 (range: 0.2-51.6). 7 of 15 surgeons averaged less than 5 RP’s per year (2 of 3 within NFTA group and 5 of 10 within the NA group). In our cohort, the majority of RP’s were performed in non-academic centers (70.1%). The median age of the study population was 62 years. The majority of patients had organ-confined disease (71.5%) with a Gleason Sum of 7 (60.5%). 3.1% of patients had positive lymph nodes on final pathology. Overall PSM rates for organ confined disease were 28.1%, 49.2% and 47.9% for FTA, NFTA and NA groups accordingly.

**Table 1:** **Patient characteristics (n = 1080)**

		Surgeon affiliation (%)			
	All groups (%)	FTA, n=238 (22.0)	NFTA, n=85 (7.9)	NA, n=757 (70.1)	p-value
Age*	62 (9.3)	60 (8.4)	62 (8.6)	64 (9.0)	<0.01
Pathological Stage					0.38
pT2	772 (71.5)	160 (67.2)	65 (76.5)	547 (72.3)	
pT3a	168 (15.6)	39 (16.4)	14 (16.5)	115 (15.2)	
pT3b	121 (11.2)	34 (14.3)	6 (7.1)	81 (10.7)	
pT4	19 (1.8)	5 (2.1)	0 (0.0)	14 (1.9)	
Gleason Sum					0.04
≤ 6	295 (27.4)	64 (26.9)	16 (19.3)	215 (28.5)	
7	653 (60.7)	147 (61.8)	63 (75.9)	443 (58.8)	
≥ 8	127 (11.8)	27 (11.3)	4 (4.8)	96 (12.7)	
Node Status					0.06
Positive	30 (3.1)	12 (5.4)	1 (1.3)	17 (2.5)	
Negative	945 (96.9)	210 (94.6)	76 (98.7)	659 (97.5)	

*Median (inter-quartile range).

On univariable analysis, age, pathological stage, Gleason score, and surgeon group were all statistically significant predictors of PSM (Table 2). Multivariable results are presented in Table 3. Surgeon volume was an independent predictor of margin positivity, with low and medium volumes being associated with lower rates of PSM (p = 0.0166). Pathological stage and Gleason sum were also independent predictors of PSM (p = 0.0004 and p < 0.0001, respectively). After controlling for surgeon volume, pathological stage and Gleason sum, the surgeon group remained independently associated with PSM (p < 0.0001). Overall, NFTA surgeons were associated with a higher rate of PSM than FTA surgeons (OR 2.5, 95% CI: 1.44 – 4.35, p = 0.001). Similarly, NA urologists were also associated with a higher rate of PSM following RP compared to the FTA group (OR 2.1, 95% CI: 1.53 – 2.88, p < 0.001). The difference between NFTA and NA urologists was not significant (OR =1.09; 95% CI 0.64 - 1.88, p = 0.492).

**Table 2:** **Univariable analysis examining predictors of positive surgical margins**

Variable	Odds ratio	95% CI	***P***-value
Age	1.02	1.00 – 1.04	0.066
Surgeon affiliation			<0.0001
FTA	Reference		
NFTA	1.83	1.11 – 3.02	
NA	1.93	1.43 – 2.60	
Pathological stage			<0.0001
pT2	Reference		
pT3a	2.36	1.67 – 3.34	
pT3b	2.08	1.41 – 3.09	
pT4	1.76	0.70 – 4.42	
Gleason score (sum)			<0.0001
≤6	Reference		
7	2.04	1.54 – 2.72	
≥8	3.46	2.24 – 5.36	
Node status			0.053
Negative	Reference		
Positive	2.09	0.97 – 4.51	
Volume of surgeries			0.113
Low (<10 cases/year)	Reference		
Med (10–20 cases/year)	0.83	0.54 – 1.28	
High (>20 cases/year)	1.10	0.73 – 1.65	

FTA: fellowship trained, academic; NFTA: non-fellowship trained, academic; NA: non-academic.

**Table 3:** **Multivariable analysis of factors predictive of positive surgical margins**

Variable	Odds ratio	95% CI	***P***-value
Surgeon affiliation			<0.0001
FTA	Reference	-	
NFTA	2.50	1.44 – 4.35	
NA	2.10	1.53 – 2.88	
Surgical volume			0.0170
High (>20 cases/year)	Reference	-	
Medium (10–20 cases/year)	0.65	0.487 – 0.878	
Low (<10 cases/year)	0.77	0.503 – 1.199	
Pathological stage			0.0004
pT2	Reference	-	
pT3a	2.08	1.45 – 2.99	
pT3b	1.63	1.06 – 2.53	
pT4	1.51	0.58 – 3.91	
Gleason score			<0.0001
≤6	Reference	-	
7	1.90	1.41 – 2.56	
≥8	2.66	1.64 – 4.31	

FTA: fellowship trained, academic; NFTA: non-fellowship trained, academic; NA: non-academic.

## Discussion

In our population-based cohort, we have shown that surgeon group was an independent predictor of obtaining PSM following RP, after adjusting for annual volume, pathological stage, and Gleason sum. RP is a complex procedure that has a steep learning curve associated with it (>250 cases) [22]. Individual surgeon experience and annual volume are important variables that have been previously shown to be associated with PSM [3–12]. Interestingly, in a small, prospective, cohort study, two newly graduated surgeons who completed formal urological oncology fellowship training at M.D. Anderson Cancer Center, showed that their results were comparable to results of RP’s performed by very experienced surgeons in larger series [21]. In this study, their first 66 consecutive patients undergoing RP were assessed from a tertiary, academic referral center. Their overall PSM rate was commendable at 14% while achieving a 94% 5-year biochemical, disease free survival rate. The author’s highlight that a strong urological residency combined with their surgically intense (approximately 87 RP’s) clinical fellowship likely enhanced their proficiency in performing RP. As a growing number of urologic oncology fellowship trained surgeons enter academia, the impact of oncology-specific fellowship training on pathological outcomes is important to address as it represents an objective means of evaluating this additional training. Certainly, the concept that specialization may improve outcomes is not novel, yet our study is the first to show in a population-based design, that urological oncology fellowship training is associated with improved rates of PSM. The reason for this is unclear however others have suggested this difference may be due to improved surgical technique or perhaps that those who undergo fellowship training are more critical of their own surgical approach [23].

In addition, there is a paucity of population-based literature examining pathological outcomes following RP and we have also shown that PSM rates are likely higher in “real-life” which may suggest that studies based out of tertiary cancer centers may not necessarily hold true at the population-based level. In another population-based study, the rate of PSM after RP in organ-confined disease was 33% [18], comparatively lower than our NFA and NA groups in our study, but higher than our FTA group. However, even amongst experienced surgeons, the rates of PSM shows considerable variability, ranging from 10-48% [7]. Many studies examining PSM rates are from tertiary cancer centers and may not truly represent the “real world” which endorses the importance of conducting population-based studies. Regardless, the rates of PSM in our study are high. The reasons for this are unclear but are likely multi-factorial. One explanation may be the significant heterogeneity within each surgeon groups. Almost half of surgeons included in this population averaged less than 5 RP’s per year (2 of 3 NFTA surgeons, and 5 of 10 NA surgeons) that may have affected outcomes, particularly given the small group sizes. Their inclusion adds variability to the results, but highlights the “real-world” urologic practice. In fact, a study based out of the UK revealed that 54% of 212 urologic surgeons performed less than 10 RP’s per year, in keeping with our results [24]. Similarly, there were four surgeons (2 NFTA and 2 NA) who stopped performing RP’s midway through the study period around the same time that the FTA surgeons volume began to increase which may suggest a generational change in practice that may be confounded by the fellowship training. Although experience is an invaluable asset in performing RP, we were unable to account for this in our analysis.

There are several limitations to our study. Firstly, a significant proportion of patients (16.9%) were excluded from our analysis due to unavailable pathological reports. The inclusion of these missing patients potentially could affect our models and outcomes. However, for a population-based study, the inclusion of over 80% of patients may also be viewed as strength, as other similar studies have drawn conclusions from a notably smaller proportion of patients [18]. Another perceived limitation of our study may be the lack of follow-up to assess disease-specific and overall survival. Data availability precluded this. However, the study was not designed to assess clinical outcomes but rather to examine differences in PSM rates. Others have already shown that PSM may be a surrogate for oncological outcomes [7, 13, 15–18]. In contrast to previously published literature, we also found that low and medium volume surgeon groups were associated with reduced PSM. This may be due to the relatively small number of surgeons practicing in this region (n = 15), making the results for each group easily influenced by a limited number of individuals. In fact, although the FTA group consisted of surgeons with moderate-high average annual RP volumes (median 21.5, range 13.4-27.6), overall 70% of RP’s were performed in non-academic centers where the average number of RP’s per year ranged from 0.2-51.6. In addition, our study did not have central pathology review. The histo-pathological interpretation of RP specimens is inherently subjective, yet although inter-observer variability exists, it has been shown that concordance between expert urological pathologists regarding PSM are excellent [25]. Others have also shown that the location, length, and Gleason sum of the PSM has prognostic significance [26], and that not all PSM carry the same risk of developing biochemical recurrence [27, 28]. Unfortunately, data constraints prevented us from assessing this. Further, we did not have data on other potentially confounding variables such as nerve-sparing status, prostate volume, tumor volume or patient factors including body-mass index or comorbidities. The majority of RP’s described within this study were by traditional open approach and may not apply to contemporary, minimally invasive techniques. Additionally, functional outcomes are another measure of successful surgery but were unable to be captured in the present study. Finally, our data apply to groups of urologists categorized by fellowship training and academic practice, and should not be extrapolated to individual surgeon performance.

Despite these limitations, our study shows that in this population-based cohort treated in the contemporary PSA era, academic surgeons with fellowship training were associated with a reduced risk of PSM. This finding highlights an important surgeon related factor that should be considered but requires further investigation in larger studies.

## Conclusion

After adjusting for pathological stage, Gleason sum and surgeon volume, RP performed by oncology fellowship trained urologists were associated with significantly lower rates of PSM. This training may provide additional knowledge and skills to shorten the learning curve associated with RP. Furthermore, our results suggest that surgeon level of training be considered in future studies examining outcomes post-RP.

## Abbreviations

RP: Radical prostatectomy
PCa: Prostate adenocarcinoma
PSA: Prostate specific antigen
PSM: Positive surgical margin
FTA: Fellowship-trained, academic
NFTA: Non-fellowship-trained, academic
NA: Non-academic.
